# S2-DyGNN: A Spectro-Spatial Dynamic Graph Neural Network for Acoustic Event Classification in Distributed Acoustic Sensing

**DOI:** 10.3390/s26144417

**Published:** 2026-07-12

**Authors:** Seunghun Jeong, Huioon Kim, Young Ho Kim, Hyoyoung Jung, Hong Kook Kim

**Affiliations:** 1 Department of AI Convergence, Gwangju Institute of Science and Technology, Gwangju 61005, Republic of Korea; zldzmfoq12@gm.gist.ac.kr; 2 Optical Precision Measurement Research Center, Korea Photonic Technology Institute, Gwangju 61007, Republic of Korea; pcandme@kopti.re.kr (H.K.); kimyh@kopti.re.kr (Y.H.K.); 3 Department of Electrical Engineering and Computer Science, Gwangju Institute of Science and Technology, Gwangju 61005, Republic of Korea

**Keywords:** distributed acoustic sensing, dynamic graph generator, event classification, graph neural network, spatial–temporal modeling

## Abstract

Distributed acoustic sensing (DAS) systems capture complex, nonlinear wave propagation across fiber-optic cables. Conventional event classification architectures, constrained by static physical topologies or isolated spatial grids, fail to effectively adapt to the dynamic feature relationships associated with such events, particularly when modeling complex spatiotemporal interactions across sensor arrays. To resolve these structural limitations, we introduce the Spectro-Spatial Dynamic Graph Neural Network (S2-DyGNN), whose architecture couples a two-dimensional frequency–time convolutional front-end with a dual-matrix graph neural network (GNN). First, the convolutional module extracts spectro-temporal features, explicitly capturing localized acoustic dynamics independent of inter-sensor interference. Subsequently, the graph module constructs a dual-matrix topology, fusing a static physical distance prior with a data-driven adjacency matrix that recalculates spatial connections frame by frame from input signals. When evaluated on a highly skewed nine-class DAS field dataset, S2-DyGNN outperformed other conventional models by achieving a peak macro-averaged F1-score of 86.6% and an overall accuracy of 94.0%. The dual-matrix graph topology prevented dominant background features from washing out sparse transient events, improving the minority “openclose” class F1-score to 55.7% compared to the 48.0% ceiling of a static graph topology. These results demonstrate that explicitly coupling localized spectro-temporal representations with physically anchored spatial topologies consistently outperforms models that process these domains in isolation, providing a highly robust and scalable solution for real-world continuous monitoring systems.

## 1. Introduction

Fiber-optic distributed acoustic sensing (DAS) has fundamentally reshaped large-scale monitoring across diverse scientific and industrial domains [1,2]. At their core, DAS interrogators operate based on phase-sensitive optical time-domain reflectometry (Φ-OTDR): when external acoustic waves or vibrations exert dynamic strain on the sensing fiber, they induce microscopic variations in the optical phase of the Rayleigh backscattered light. By converting standard glass optical fibers into continuous arrays of vibration sensors through this precise phase demodulation, DAS can capture acoustic events along a 50 km pipeline, a busy highway, or even submarine telecommunication cables to track ships and earthquakes [3,4]. When a single optical cable is transformed into tens of thousands of virtual sensors, it produces a highly detailed acoustic map of the surrounding environment; however, the sheer scale of uninterrupted data produced by such systems makes robust automated pipelines indispensable [5]. Beyond the sheer size of the data, real-world signals are often noisy and irregular [6]. In subterranean deployments, signal attenuation caused by soil and elevated ambient noise frequently results in a low signal-to-noise ratio (SNR) [7,8], which complicates accurate event classification. Consequently, the field is rapidly shifting toward machine learning architectures [9,10].

Early event classification approaches employing DAS relied heavily on manual feature engineering to develop machine learning methods [11], extracting and feeding handcrafted signal features into standard machine learning classifiers [2,12]. Building on this foundation, subsequent studies sought to address specific operational bottlenecks: to improve signal clarity, some utilized variational mode decomposition (VMD) to separate valid signals from background noise [13], and other approaches processed data directly in the compressed domain to tackle the massive storage requirements of DAS [14]. Although these handcrafted features provided an initial framework, they proved fragile in practice [15]. Conventional frequency filters typically require extensive site-specific manual calibration [16]; however, they remain highly vulnerable to performance degradation under fluctuating ground conditions or the emergence of unanticipated background noises [17]. Such nonadaptive signal processing formulations fail to accommodate the unpredictable nature of field vibrations.

Currently, convolutional neural networks (CNNs) are extensively employed to extract features directly from DAS signals; for example, one-dimensional (1D) CNNs are actively deployed to extract discrete vehicle signatures [18], whereas two-dimensional (2D) CNNs capture the spatiotemporal propagation of acoustic waves across continuous multichannel arrays [19]. However, standard CNNs impose structural constraints by treating the sensor array as a rigid grid [20], and this structural dependence on fixed-size windows restricts the network to defining sensor interactions purely through static spatial distance [21]. In real-world scenarios, acoustic wave propagation across fiber-optic cables rarely exhibits homogeneous spatial dispersion, as the spatial footprint of an event fluctuates drastically with source magnitude: a low-energy transient may excite only a few adjacent channels, whereas a high-impact disturbance can simultaneously propagate across dozens of sensors. Rigid convolutional grids, constrained by static receptive fields, fail to adapt to such highly variable spatial scales. Beyond this, the reliability of the DAS signals is often compromised by the unavoidable spatial attenuation and random phase fading occurring along the fiber [22,23]. Consequently, rigid fixed-size filters and static graph topologies prove inadequate when confronted with instantaneous physical variations. To resolve these structural limitations, we propose the Spectro-Spatial Dynamic Graph Neural Network (S2-DyGNN), which processes multisensor data through a two-stage pipeline: localized spectro-temporal feature extraction followed by adaptive spatial aggregation.

Before feature extraction, a short-time Fourier transform (STFT) converts 1D acoustic time series into isolated spectrograms. The architecture then employs a 2D frequency–time (FT) convolutional front-end. The system evaluates each physical sensor as an independent spatial channel, preserving intra-channel acoustic dynamics and preventing premature spatial aggregation. The STFT output establishes a precise 2D grid of discrete time steps and frequency bins. Retaining this original spectro-temporal layout forces the network to track pitch shifts and localized acoustic energy. A series of asymmetric convolutions progressively compresses the spectral dimensions across this input grid to extract high-level frequency signatures. Importantly, these specific filters preserve the original temporal resolution, and maintaining exact time-axis alignment prevents brief transient anomalies from being temporally smeared. The network ultimately converts the raw continuous spectrograms into dense, node-specific feature sequences, which we then use as the baseline node embeddings for dynamic aggregation.

The graph neural network (GNN) then integrates these localized signatures to capture the spatial dependencies of acoustic features along the fiber. We adopt this graph-oriented approach specifically because real-world acoustic waves naturally defy rigid spatial grids. Rather than relying solely on dynamic attention or static physical topologies, the proposed system fuses both, embedding the fixed physical distances among sensors as a baseline static matrix to reflect natural wave attenuation. The network then dynamically updates these connections frame by frame using an attention mechanism driven by real-time spectral similarities. The fused adjacency matrix restricts feature aggregation to correlated sensor nodes, isolating uninformative channels computationally by reducing their connection weights toward zero. Extensive experiments on field DAS datasets demonstrate that this targeted spatial aggregation yields exceptional classification performance while maintaining low computational latency. By achieving this optimal balance, S2-DyGNN offers a highly scalable, ready-to-deploy solution for critical edge-computing applications such as real-time pipeline leak detection, railway monitoring, and perimeter security.

The main contributions of this study are as follows:

- Independent feature extraction: The system processes each sensor node separately rather than analyzing the entire array simultaneously. Data from a single node form a simple 2D grid of time and frequency, and the CNN compresses the frequency information while retaining the full temporal resolution. Rather than building the global structure immediately, we first extract the localized acoustic signatures to anchor the broader graph.
- Event-driven dynamic spatial aggregation: The graph topology fluidly reconfigures to mirror the input data. An attention module actively computes spectral similarities to form new edges, allowing the dynamic graph to capture localized vibration patterns, and a static distance matrix is then applied as a supplemental boundary. This fusion stabilizes the generated graphs.
- Structural resistance to signal washout: The attention mechanism in the proposed GNN explicitly suppresses connections to purely noisy nodes. This adaptive spatial filtering prevents continuous background noise from averaging out localized transients. Consequently, the system maintains classification accuracy for highly sparse, short-duration occurrences even under severe field-data imbalance.

The remainder of this paper is structured as follows: Section 2 reviews related work to identify the limitations of isolated processing models; Section 3 introduces the underlying DAS physics, while Section 4 details the data collection process; Section 5 presents the S2-DyGNN architecture, with particular emphasis on the handoff between the 2D FT CNN and dual-matrix dynamic GNN; Section 6 defines the experimental environment and metrics; Section 7 reports the quantitative results and ablation studies that confirm the effectiveness of S2-DyGNN; and Section 8 concludes the paper.

## 2. Related Work

Pattern recognition methods for DAS have evolved from conventional feature engineering and machine learning to deep learning techniques that better capture complex acoustic signatures. Convolutional, recurrent, and early graph-based models have each contributed to important advances in processing continuous fiber-optic data; nevertheless, many of these methods either introduce severe temporal computational bottlenecks or impose rigid constraints on the spatial relationships between vital sensor channels. This section reviews these evolutionary steps and underscores the need for architecture that adapts to both spatial and temporal dependencies.

### 2.1. Conventional Feature Engineering and Machine Learning in DAS

Initial DAS classification systems relied almost entirely on manual signal processing. The fiber-optic array was typically treated as a series of independent acoustic channels, with STFT or basic wavelets applied to extract frequency signatures [3,11]. Given the intense attenuation and complex noise environments inherent to buried cables, standalone filtering approaches consistently fall short in real-world deployments. Accordingly, researchers constructed complex hybrid pipelines to deal with severely degraded SNRs; for example, transient metrics such as skewness and kurtosis were extracted using VMD and higher-order statistics as a workaround for support vector machine classification [13], and Kapoor et al. [14] reduced computational overhead by extracting features directly from compressed files, relying exclusively on predefined physical signatures. The immense data load of continuous DAS monitoring essentially forces these operational compromises. Furthermore, principal component analysis [24] and wavelet packet decomposition [10] have become routine preprocessing steps to clean raw acoustic data before applying any machine learning.

This heavy reliance on manual feature extraction creates a severe generalization bottleneck, as engineers must tightly tune mathematical filters to localized physical parameters, such as specific vehicle weight classes or regional soil mechanics. The critical limitation of these methods is environmental variability: mathematical filters calibrated for specific weather or soil conditions degrade immediately upon changing the physical site, meaning that these models are prone to overfitting on the initial calibration data. Consequently, conventional pipelines lack the adaptability required to survive unpredictable, long-term monitoring.

### 2.2. Conventional Deep Learning in DAS and Its Limitations

Moving beyond manual feature engineering, recent studies have increasingly aligned network designs directly with the physical structure of DAS signals. In vehicle detection, Chiang et al. [18] successfully isolated discrete acoustic events by applying 1D CNNs along individual spatial channels. Although this 1D approach is highly efficient for localized signatures, it naturally misses the broader context of wave propagation; to address this limitation, multichannel DAS data are often formatted as a continuous spatiotemporal grid for 2D convolutional models. Liu et al. [19] adopted this 2D strategy for microseismic data, demonstrating that spatial receptive fields are well suited to tracing physical wavefronts propagating across adjacent optical sensors.

In addition to spatial modeling, modeling the temporal dynamics of these acoustic events is equally critical. Stacking convolutional extractors with recurrent modules, particularly long short-term memory (LSTM) units, is a common strategy, one used by Wang et al. [25] to build a hybrid CNN-LSTM setup for Φ-OTDR pattern recognition. Kayan et al. [26] applied a similar logic, combining deep transfer learning with recurrent neural networks to process phase-stacked sequences. Despite their strong sequential-modeling capabilities, recurrent networks suffer from a fundamental architectural flaw: they must process data step by step, which introduces a severe computational bottleneck that prevents parallelization. Given the massive, high-frequency continuous data streams generated by DAS monitoring, the recursive parameter overhead and inherent inference latency of LSTMs make them impractical for real-time edge deployment. Consequently, there is a pressing need for spatial–temporal architectures that can model dynamic feature relationships without sacrificing parallel-processing efficiency.

Standard CNNs also face a fundamental structural limit: they evaluate the sensor array as a rigid grid. However, acoustic wave propagation rarely exhibits homogeneous spatial dispersion, as the spatial footprint of an event varies drastically with source magnitude: a low-energy transient may excite only a few adjacent channels, whereas a high-impact disturbance propagates across dozens of sensors simultaneously. A static convolutional window cannot resize dynamically, making it impossible to adapt to feature patterns that continually shift in spatial scale.

### 2.3. Spatial Modeling Using Graph Neural Networks

By formulating the optical-fiber sensors as independent graph nodes, GNNs sidestep the spatial rigidity of standard CNNs, thereby unlocking a far more flexible spatial representation [27]. Shahabudin et al. [28] applied graph topologies to detect microseismic events directly from DAS outputs. Despite these structural advancements, integrating DAS data into current graph pipelines faces two critical bottlenecks: First, many existing architectures feed raw 1D time-series data directly into network nodes. By bypassing spectral decomposition and early convolutional encoding, these models fail to extract rich, localized frequency features before message passing. Second, conventional graph convolutional networks (GCNs) rely on static adjacency matrices defined solely by the physical distance between sensors. As a fixed-topology graph cannot dynamically reconfigure its internal pathways, it fails to adapt to the irregular and rapidly shifting spatial footprints of transient acoustic vibrations. Bridging this critical gap requires a new architectural paradigm. The S2-DyGNN architecture directly addresses these bottlenecks by coupling a temporal-preserving 2D CNN front-end (i.e., preserving temporal alignment of features within the network) with a data-driven dynamic graph-generation mechanism. The core edge formulation is guided by a data-driven attention mechanism that establishes edges on the basis of immediate acoustic correlations. To stabilize this dynamic rewiring, predefined physical distances are incorporated as a baseline spatial constraint, ensuring that the generated graph topology incorporates physical distance priors while effectively capturing the transient feature footprints of the events.

## 3. Principles of the DAS System

The architectural design of the proposed neural network is directly informed by the physical data-acquisition process of the DAS hardware. This section explains the optical principles, system parameters, and the physical nature of the resulting spatiotemporal signals [27].

### 3.1. Principles of Phase-Sensitive OTDR

Data acquisition relies on Φ-OTDR. In this setup, a standard optical fiber functions as a massive, continuous sensor array [29], and an interrogator transmits highly coherent laser pulses through the glass core of the fiber. During optical signal propagation, microscopic variations in the refractive index of silica continuously scatter a small fraction of the light backward, a phenomenon called Rayleigh backscattering. As nearby ground vibrations sweep across the buried cable, the impinging acoustic waves physically perturb the optical fiber, and the resulting dynamic strain subtly alters both the geometric length and the refractive index of the silica core. Such localized changes shift the optical phase of the backscattered signal, and continuous measurement of these phase differences enables the system to localize acoustic events in both time and space.

### 3.2. System Parameters and Mathematical Relationships

The physical settings of the hardware determine the structure of DAS data before they reach the neural network. The raw output forms a massive 2D grid. Mapping the acoustic stream onto a spatiotemporal grid discretizes the fiber into a continuous array of virtual sensors. The physical spacing between adjacent channels, termed the spatial sampling distance (Δz), is dictated by the system’s sampling interval and the velocity of backscattered light, as shown in Equation (1):

(1) $$\Delta z=\frac{c_{0}\cdot t_{s}}{2n},$$

where $c_{0}$ is the speed of light in vacuum, $t_{s}$ is the sampling interval, and *n* is the refractive index of the glass core (typically ~1.468). Division by two accounts for the laser pulse traveling down the fiber and reflecting back. The temporal sampling distance, Δt, is directly related to the pulse repetition rate, $f_{p}=1/\Delta t$, of the interrogating laser.

Another critical parameter is the gauge length ($L_{g}$), which defines the actual physical span of fiber that detects vibrations, in contrast to Δz, which denotes only the gap between sensors. The former depends directly on the laser pulse width (τ):

(2) $$L_{g}=\frac{c\cdot \tau }{2},$$

where *c* denotes the speed of light in an optical fiber (typically $c=c_{0}/n\approx 3\times 10^{8}/1.468\approx 2.04\times 10^{8}$ m/s). This introduces a physical trade-off: short pulses provide precise localization of acoustic events, whereas long pulses increase sensitivity to weak signals. In practice, utilizing pulse widths of 10 to 100 ns resolves the competing demands of signal sensitivity and spatial acuity.

### 3.3. Physical Interpretation of Acoustic Signatures

The operational basis of DAS relies on tracking optical phase shifts (Δϕ). As acoustic waves permeate the ground, they mechanically couple with the buried cable, causing microscopic elongations and compressions. This applied strain shifts the phase of the backscattered signal, an opto-mechanical relationship formulated by the photoelastic effect:

(3) $$\Delta \phi =\frac{4\pi n}{\lambda }\xi L_{g}\Delta \varepsilon \left(x,t\right).$$

In this formula, λ denotes the laser wavelength, and ξ characterizes the photoelastic coefficient of the fiber. The crucial variable here is $\Delta \varepsilon \left(x,t\right)$, which quantifies the actual strain on the cable at a specific location *x* and time *t*. Because the cable deforms in direct response to surrounding vibrations, calculating this phase shift effectively transforms the entire optical fiber into a highly sensitive acoustic sensor. Because the data were acquired using a commercial interrogator operating at a high 20 kHz sampling rate, its internal optical demodulation and phase unwrapping suite effectively mitigates modulo-2π ambiguity for the targeted acoustic events. Consequently, the extracted 1D acoustic signals are continuous, requiring no further phase reconstruction in our preprocessing pipeline.

### 3.4. Characteristics of DAS Data for Pattern Recognition

From a machine learning perspective, Equations (1)–(3) illustrate why raw DAS data is particularly challenging to process: the system outputs a massive, continuous 2D matrix of phase values. Because acoustic waves ripple through the ground and hit the fiber over a physical area, a single target event is rarely confined to one spatial sampling point; instead, the strain Δε extends across several adjacent channels. For example, a heavy excavator will create an immense spatial footprint spanning dozens of nodes, whereas a single footstep may only register on two or three. Furthermore, the true signal is continually masked by heavy environmental background noise, and standard pattern recognition systems that attempt to view such dynamic, noisy data through fixed-size windows often fail to capture the exact physical extent of an event, underscoring the need for a more adaptable, graph-based modeling approach [27].

## 4. Data Collection

This section outlines the data collection process used to assemble the dataset for evaluating the proposed event classification model. We employed a large-scale DAS dataset [30] encompassing both routine environmental noises and simulated situations. The following subsections describe the physical testbed setup, the categories of recorded acoustic events, and the manual labeling steps required to prepare the data for model training.

### 4.1. Site Configuration and Installation

Data were collected using a Φ-OTDR system. A standard ITU-T G.652.D [31] single-mode fiber, buried 1 m underground alongside a university campus pavement, was used as the sensing medium, and the interrogator operated with a 1550 nm laser, pulse repetition rate of 20 kHz, and pulse width of 20 ns. This setup provided a spatial sampling interval of ~1.02 m, resulting in 1663 sensing points along the fiber. Table 1 summarizes these operational parameters for the DAS system.

### 4.2. Event Categories

The collected dataset contains various types of vibrations, covering routine operational events such as pedestrian walking, running, vehicle traffic, and longboard usage, as well as specific security-related scenarios, including fence climbing, cable manipulation, and opening or closing of manhole covers. Table 2 lists the event scenarios and the data associated with each scenario during data collection.

### 4.3. Data Structure and Labeling

**Figure 1:**
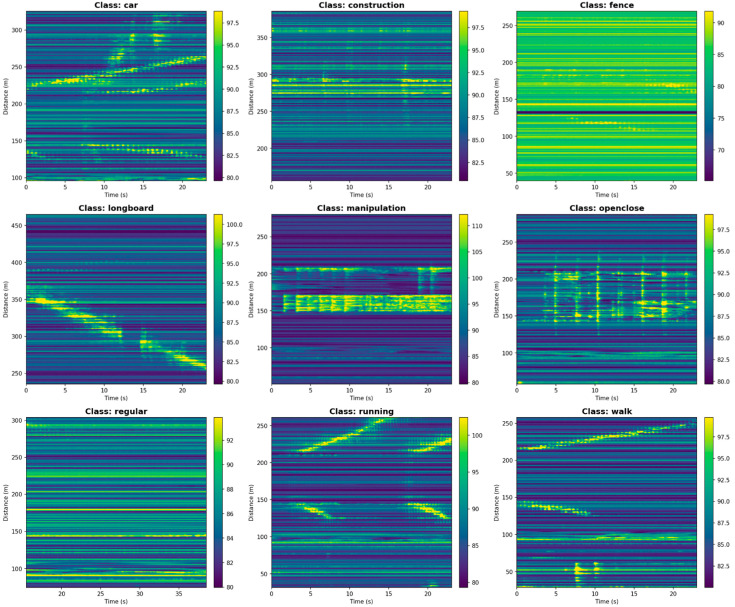
Spatial–temporal waterfall plot of the band-limited signal power for all nine event classes.

Raw 1D acoustic signals, corresponding to optical phase variations induced by acoustic disturbances, were extracted from sensing points and stored in HDF5 format. After the DAS data was fully recorded, the entire dataset was manually annotated by a single expert to eliminate inter-annotator variability. Rather than individually reviewing each of the 324,000+ segmented windows, the labeling process was conducted at the continuous recording-session level by evaluating the band-limited signal power ($P_{band}$) within the 50–500 Hz frequency range. To illustrate the visual separability of the classification task that guided this annotation, representative spatial–temporal plots of $P_{band}$ for all nine event classes are presented in Figure 1. The assigned labels were subsequently inherited by all corresponding segments during the windowing process. Crucially, the labeling process utilized the exact same sliding window parameters as the data segmentation, which ensures that the generated label boundaries align perfectly with the extracted data, allowing the session-level annotations to be directly and accurately inherited by all corresponding segments. For feature extraction and model training, the continuous 1D signals were segmented into discrete blocks using a sliding window approach with a 2048-sample shift, with each block containing 8192 samples. This framing strategy directly determines the input dimensions for the proposed feature encoder.

## 5. S2-DyGNN for DAS Event Classification

**Figure 2:**
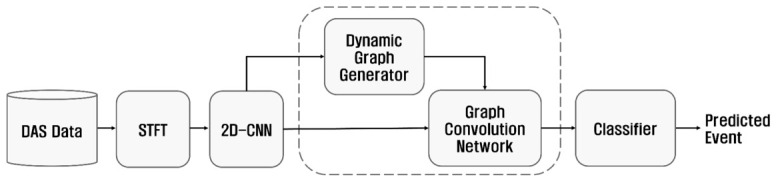
Structure of the proposed S2-DyGNN model.

**Figure 3:**
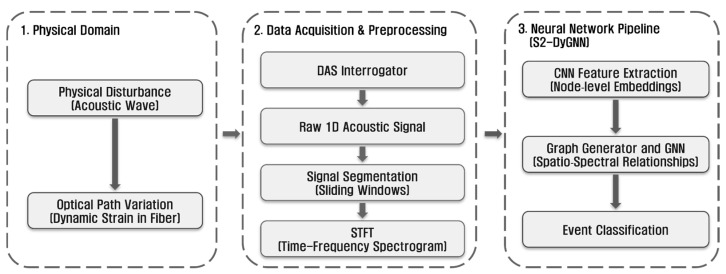
Overall data-flow pipeline.

This section introduces the S2-DyGNN developed for event classification on the DAS dataset. The processing pipeline proceeds in four stages (Figure 2): first, the raw DAS data are processed using STFT; second, a 2D CNN extracts independent features from the time–frequency grids; third, a dual-matrix graph module, comprising a dynamic graph generator and a GNN, processes spatial interactions across multiple sensors; finally, the classifier outputs an event class from the features. The following subsections describe each component. To clarify the transition from physical measurements to the neural network input, the overall data-flow pipeline can be described as shown in Figure 3. Initially, a physical disturbance occurring near the sensing fiber induces a dynamic strain, causing optical path variations within the fiber. These variations are detected by the DAS interrogator, which outputs a corresponding raw 1D acoustic time signal. To process this continuous data, the system first segments the signal into discrete windows and then transforms them into time–frequency spectrograms via the STFT. Subsequently, a CNN extracts high-level feature embeddings from these spectrograms. Finally, these node-level features are fed into the dynamic graph-generation module, which constructs the spatio-spectral relationships necessary for the final event classification.

### 5.1. Spectro-Temporal Feature Extraction Using Asymmetrical 2D CNN

Raw DAS measurements often exhibit low-frequency baseline shifts and phase noise [32,33]. To mitigate these effects, the 1D acoustic signals are first converted into 2D time–frequency grids, and STFT is applied with a window size of 1024 and a hop length of 256. This conversion yields a spectrogram matrix $X_{i}\in R^{T\times F}$ per sensor node *i*, structured across *T* temporal frames and *F* = 513 frequency bins. This 2D format provides a localized time–frequency representation of the signal across specific frequency bands, making the acoustic patterns easier to classify compared to those in raw waveforms [34].

**Figure 4:**
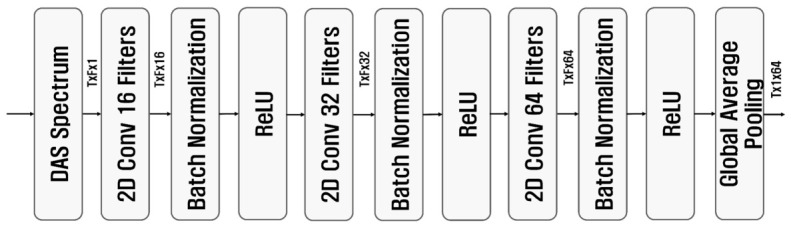
Structure of the frequency–time 2D CNN.

As illustrated in Figure 4, a 2D CNN is employed to extract localized features from these grids within the time–frequency domain. Standard 2D CNNs typically downsample both axes equally; however, our system analyzes time-varying feature relationships across multiple sensors over time, which may reflect the spatial patterns caused by acoustic wave propagation. This task requires precise temporal alignment between channels: if a model downsamples the time axis, it loses the exact timing of the wave arrival at each sensor. Therefore, we designed an asymmetrical CNN front-end to extract features without losing temporal precision. The encoder stacks three sequential convolutional blocks, and the channel depth increases progressively from 16 to 32, and finally to 64. Each layer uses an asymmetrical kernel size of 3 × 5 and an asymmetrical stride of 1 × 2; a stride of 1 along the time axis preserves the temporal resolution frame by frame, and a stride of 2 along the frequency axis steadily downsamples the spectral information. Batch normalization (BN) [35] and rectified linear unit (ReLU) activation [36] follow each convolution to stabilize training.

After the final convolutional block, an adaptive average pooling layer is applied, which compresses only the frequency dimension to size 1 while preserving the temporal dimension. The subsequent graph-generation module then compares features across sensors at identical time frames, ensuring temporal alignment, and a squeeze operation then removes the redundant frequency dimension, yielding the mapping function $f_{\mathrm{CNN}}$:

(4) $$H_{i}=f_{\mathrm{CNN}}\left(X_{i}\right)\in R^{d\times T},$$

where $H_{i}$ is the output feature sequence for node *i*, and *d* = 64 is the hidden channel dimension. This architectural design successfully extracts intra-node spectro-temporal embeddings from the time–frequency domain while strictly preserving the original temporal sequence for the dynamic graph generation.

### 5.2. Data-Driven Spatial Aggregation via Dual-Matrix Formulation

**Figure 5:**
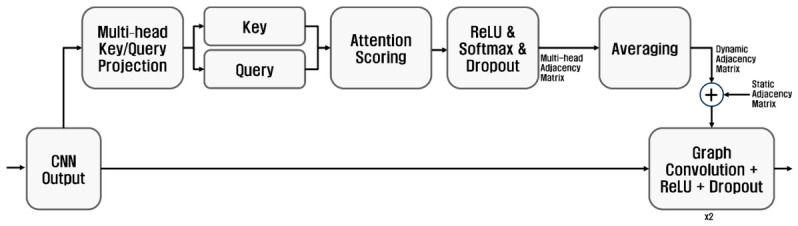
Structure of the graph generator and GNNs in the S2-DyGNN model.

After extracting the independent time–frequency features, the network analyzes data-driven dependencies between spatial nodes to capture feature patterns associated with acoustic wave propagation along the optical fiber. To capture these wave-propagation interactions across sensor nodes along the fiber, we designed a data-driven graph generator module. Figure 5 illustrates the internal data flow of this module, which comprises a graph generator and a GNN [37]. The CNN output features serve as inputs to both components. The graph generator projects the inputs into multi-head keys and queries to compute initial attention scores. Rather than using these scores directly, the module fuses the dynamic attention with a static, distance-based prior, imposing a physical constraint that penalizes highly distant, improbable connections. Finally, two stacked graph convolution blocks apply this hybrid matrix to aggregate the CNN features.

#### 5.2.1. Limitations of Static Topologies

Conventional GNNs [38] connect nodes using fixed edges. Owing to their typical reliance on static values such as the physical distance between sensors, adjacent channels receive the highest connection weights, while distant channels are assigned weights close to zero. This approach assumes ideal conditions in which sound waves propagate perfectly and symmetrically along the fiber; however, in actual field deployments, the acoustic landscape grows vastly more complex, as wave propagation is continually skewed by heterogeneous soil profiles, diverse cable packaging, and concurrent background noise [8]. Fixed graphs use fixed inter-node relationships and therefore cannot adapt their graph topology to time-varying feature relationships in the measured data. Therefore, instead of relying on a fixed distance formula, the proposed graph generator module computes a new adjacency matrix for each data frame, meaning that the network continuously reconfigures its graph topology to adapt to time-varying feature relationships extracted from the measurements.

#### 5.2.2. Multi-Head Attention for Dynamic Adjacency Matrix Generation

To compute the dynamic edge weights, we utilize a multi-head self-attention mechanism (Figure 5). For any given temporal frame $t\in \left[1,T\right]$, we denote the corresponding node feature matrix as $H(t)\in R^{N\times d}$, where *N* represents a local neighborhood of sensors and d=64 is the hidden dimension extracted by the CNN front-end. For each attention head *k*, the model linearly projects node features into query ($Q_{k}$) and key ($K_{k}$) matrices at time *t*:

(5) $$Q_{k}(t)=H{(t)W}_{Q}^{\left(k\right)},\;\;K_{k}(t)=H(t)W_{K}^{\left(k\right)},$$

where $W_{Q}^{\left(k\right)}$ and $W_{K}^{\left(k\right)}$ are learnable weight matrices. Raw correlation scores $S_{k}\left(t\right)$ between sensors are then computed by a scaled dot-product operation:

(6) $$S_{k}(t)=\frac{Q_{k}(t)K_{k}^{T}(t)}{\sqrt{d_{k}}}.$$

To obtain non-negative attention scores before normalization, ReLU is applied as an architectural constraint rather than a direct physical rule, suppressing weak or unreliable links; therefore, the softmax function is not applied directly to these raw scores. First, ReLU activation is applied to force all negative values to zero, a physical sparsification step that eliminates irrelevant or noisy sensor links. By applying a softmax operator across the node dimension, we constrain the positive weights $A_{k}(t)$ into a valid attention distribution as follows:

(7) $$A_{k}(t)=\mathrm{Softmax}\left(\mathrm{ReLU}\left(S_{k}(t)\right)\right).$$

Our architecture uses two independent attention heads, each capable of learning diverse feature relationships, such as those associated with the primary event source or propagating patterns. Their outputs are averaged as follows to finally form a stable dynamic adjacency matrix for that specific time step:

(8) $$A_{dyn}\left(t\right)=\frac{1}{2}\sum_{k=1}^{2}A_{k}(t).$$

Nevertheless, relying exclusively on this data-driven attention poses a structural risk of overfitting to spurious ambient noise. Accordingly, we introduce a static distance matrix, $A_{sta}$, to constrain this behavior, which maps the actual physical separation of the fiber sensors, quantitatively reflecting baseline wave attenuation. We define the overall adjacency matrix $\tilde{A}\left(t\right)$ by taking a convex combination of the dynamic and static graphs as follows:

(9) $$\tilde{A}\left(t\right)=\left(1-\alpha \right)A_{dyn}\left(t\right)+\alpha A_{sta},$$

with $\alpha \in \left[0,1\right]$ acting as a penalty term for physical constraint enforcement. Fusing these matrices ensures that the model can chase rapid, localized transients without ignoring the actual physical layout of the fiber.

#### 5.2.3. Spatial Feature Aggregation Using GCN

Once the final adjacency matrix $\tilde{A}\left(t\right)$ is obtained, node features are updated using GCN blocks, and two identical GCN layers are stacked to expand the receptive field across the sensor neighborhood. At each layer *l*, the network aggregates information from the dynamically connected sensors by multiplying $\tilde{A}\left(t\right)$ with the node features $H^{\left(l\right)}\left(t\right)$. The aggregated node features undergo linear projection (*W*), BN, and ReLU activation. We finalize the layer computation by applying dropout [39] and injecting a skip connection to form the updated representation as follows:

(10) Hl+1(t)=DropoutReLUBNA~(t)Hl(t)Wl+Hl(t),

with $W^{\left(l\right)}$ defining the layer-specific weights and $H^{\left(l+1\right)}(t)$ representing the updated feature matrix at time frame *t* for layer *l*+1. This additive path serves a dual purpose: it makes the learning process resistant to vanishing gradients and shields the local sensor data from structural oversmoothing. Through this mechanism, the model naturally produces a spatiotemporal signature that aligns with actual physical propagation.

## 6. Experimental Setup

We evaluated the proposed S2-DyGNN using a field-recorded DAS dataset. The experimental framework tests the model’s ability to classify localized vibrations. The following subsections describe the processing of the dataset, the specific hardware and software configurations for model training, comparative models, and the evaluation metrics used to quantify the final classification performance.

### 6.1. Dataset

An optical interrogator captured acoustic phase variations along a deployed fiber-optic cable at a sampling rate of 20 kHz. The recorded data encompassed nine distinct vibration classes. Continuous temporal streams were segmented using a sliding window of 8192 samples and a shift length of 2048 samples, and each extracted segment was transformed via STFT with a window size of 1024 and a hop length of 256, producing 513×T 2D spectrograms. To prevent data leakage, we chronologically allocated the first 80% of the extracted segments to train the network, while the validation and test phases each received 10% of the subsequent data, separated by a 5-window safety margin.

### 6.2. Implementation Details

All model training and evaluation computational experiments were conducted on an NVIDIA RTX A6000 GPU with CUDA support using the PyTorch version 1.13.1 framework. To capture local wave propagation, the spatial input was structured by grouping seventeen adjacent sensor channels; this topology comprised one central target node and four physical neighbors on each side. The hyperparameter α was set to 0.15, which was empirically chosen based on preliminary observations to provide a stable balance between data-driven adaptation and physical distance regularization. This configuration maintained a proper balance between data-driven flexibility and physical constraints. The network was trained using the AdamW optimizer [40] with an initial learning rate of $2\times 10^{-4}$ and a batch size of 128. A standard cross-entropy loss function guided the multiclass classification, and training relied on a cross-entropy loss formulation for a maximum of 30 epochs. We managed the learning rate by halving it after a three-epoch accuracy stagnation, alongside a 10-epoch early stopping patience based on validation loss.

### 6.3. Comparative Models

We benchmark the proposed S2-DyGNN against four established models, which represent different approaches to spatial and temporal feature extraction. The baseline CNN [5] and CNN-LSTM [41] were chosen as convolutional baselines, reflecting standard methods for frequency and temporal feature extraction. The basic CNN [5] processes DAS data as independent 1D signals and extracts local frequency patterns using standard convolutional filters, ignoring both temporal continuity and inter-node physical relationships. CNN-LSTM adds recurrent layers to the CNN feature extractor, capturing the temporal evolution of acoustic events within individual channels; however, it still treats neighboring sensors as isolated data streams. In addition, we evaluated two pure GNNs: GCN [28] and the graph attention network (GAT) [28]; the former aggregates features from neighboring nodes using a symmetric adjacency matrix, while the latter assigns different importance weights to neighboring nodes during aggregation. By testing these spatial-only frameworks, we deliberately highlight why spatial topology alone falls short in the absence of a dedicated sequential extraction mechanism. To ensure a rigorous and equitable comparison, input data formats and training hyperparameters were standardized across all models to strictly match those of the proposed framework.

### 6.4. Evaluation Metrics

The models were evaluated quantitatively using accuracy, precision, recall, and the macro-averaged F1-score. When c∈{1, …, 9} represents the target class and $TP_{c}$, $FP_{c}$, and $FN_{c}$ represent the number of true positives, false positives, and false negatives, respectively, the following relationship is derived:

(11) $$\mathrm{Accuracy}=\frac{\sum_{c=1}^{C}TP_{c}}{N},$$

(12) $${\mathrm{Precision}}_{c}=\frac{TP_{c}}{TP_{c}+FP_{c}},{\mathrm{Recall}}_{c}=\frac{TP_{c}}{TP_{c}+FN_{c}}.$$

Field recordings often contain imbalanced event distributions. We accounted for this using the macro-averaged F1-score, which computes the harmonic mean of precision and recall for each class and then averages them, ensuring equal evaluation of all vibration types:

(13) $${F1}_{c}=2\times \frac{{\mathrm{Precision}}_{c}\times {\mathrm{Recall}}_{c}}{{\mathrm{Precision}}_{c}+{\mathrm{Recall}}_{c}},$$

(14) $$\mathrm{Macro}\text{-}\mathrm{averaged}\;F1=\frac{1}{C}\sum_{c=1}^{C}{F1}_{c}.$$

## 7. Performance Evaluation

In the following evaluations, we benchmark S2-DyGNN against a spectrum of conventional convolutional, recurrent, and purely graph-based architectures using a nine-class DAS acoustic dataset. Beyond standard comparative metrics, we conduct a targeted ablation study to isolate the impact of both the underlying graph topology and the specific convolutional front-end. Ultimately, these quantitative assessments confirm that the proposed model more effectively captures the intricate spatial, temporal, and spectral dependencies fundamental to DAS event classification. To verify the statistical significance of the reported results, we evaluated the proposed S2-DyGNN over three independent runs with different random seeds. The standard deviations of the overall accuracy and the macro-averaged F1-score were consistently low at 0.2% and 0.3%, respectively, confirming that the observed performance variations in the results are statistically significant and not attributable to random run-to-run noise.

### 7.1. Performance Comparison with Other Models

S2-DyGNN and the comparative models were evaluated using the DAS acoustic dataset, which comprised nine classes. Quantitative performance was measured using overall accuracy and macro-averaged F1-scores. While accuracy reflects overall prediction accuracy, raw field data exhibit extreme class imbalance; therefore, macro-averaged F1-scores were used as the primary evaluation metric, as they prevent the dominant “regular” class from inflating the reported performance. S2-DyGNN was systematically compared with baseline CNN [5], CNN-LSTM [41], GAT [28], and GCN [28] models.

Table 3 compares evaluation metrics across these approaches. The baseline CNN achieved an accuracy of 84.3% and a macro-averaged F1-score of 64.1% because it processes the frequency sequence of each sensing point independently, and thus cannot model the inter-node dependencies associated with sound waves traveling along fiber-optic cables or their temporal evolution. Integrating temporal modeling via CNN-LSTM improved the accuracy to 86.0% and the macro-averaged F1-score to 71.4% because the LSTM layer captures node-specific temporal changes, but inter-node relationships remain unmodeled. Processing sensing points independently degraded the detection of rare physical features, resulting in an “openclose” F1-score of 31.2%.

Original graph models, specifically GAT and GCN, demonstrated improvements in macro-averaged F1-scores over the baseline CNN, achieving up to 75.5% with the GCN model; however, their overall performance remained limited compared to the proposed architecture. Thus, relying solely on spatial relationships without robust spatiotemporal feature extraction is insufficient for complex DAS signals. S2-DyGNN resolves these limitations by capturing both intra-channel temporal dynamics and inter-channel spatial wave propagation, thus achieving a peak accuracy of 94.0% and a macro-averaged F1-score of 86.6%. The integration of spectral–temporal analysis and inter-channel spatial information effectively isolates the effects of sparse transient impacts. Notably, the F1-score for the “openclose” event increased to 55.7%, directly validating the efficacy of the physically regularized dynamic graph.

### 7.2. Impact of Graph Topology Type

Ablation studies were conducted to isolate the effects of graph topology strategies. Table 4 reports performance variations across graph-generation mechanisms. A static GNN-CNN applying a fixed distance matrix was set as the baseline, which defines network edges based on physical distances of optical cables. While it achieved an overall accuracy of 89.9% and a macro-averaged F1-score of 78.3%, the pre-set edge weights showed limitations in capturing short anomalies; for instance, the F1-score for the sparse “openclose” events remained restricted to 48.0%. Processing all time frames with identical spatial rules hinders the network’s ability to adapt to sudden, localized changes in inter-node relationships.

Dynamic GNN-CNN replaces a fixed topology with a purely data-driven attention mechanism, recalculating edge weights for each acoustic frame. Through adaptive message passing, the overall accuracy improved to 90.6% and the macro-averaged F1-score to 85.5%. The “openclose” F1-score increased to 52.3%, indicating an enhanced, albeit still limited, focus on brief transient flows. However, this purely dynamic architecture does not consider physical boundaries; thus, the unconstrained attention mechanism can overfit distant noisy sensor channels. S2-DyGNN resolves this vulnerability by fusing the dynamic spatial graph with a static physical prior; this hybrid formulation pushed the overall accuracy to a peak of 94.0% and the macro-averaged F1-score to 86.6%. The integration of the physical distance matrix explicitly penalizes spurious spatial connections, and this structural regularization prevents attention drift toward background noise. Despite the inherent difficulty of classifying such sparse and brief events, this dual-matrix architecture achieved the highest F1-score of 55.7% for the “openclose” class among the tested topologies. This meaningful relative improvement demonstrates that the updated node embeddings better preserve raw transient signatures compared to the original fixed or dynamic topologies.

### 7.3. Impact of Convolutional Front-End Architectures

Ablation studies were also conducted to isolate the performance contribution of the convolutional front-end. Table 5 summarizes performance variations across different convolutional strategies. The structural differences dictate the quality of localized embeddings before the occurrence of graph aggregation. A 1D FT CNN restricts the receptive field to a single axis, forcing the network to ignore the joint spectro-temporal feature of the acoustic wave. Fragmented, single-axis feature extraction fails to isolate weak transient signals from ambient background noise; consequently, this variant recorded an overall accuracy of 88.6% and a macro-averaged F1-score of 68.4%. The minority class metrics expose the exact failure point: the F1-score for the sparse “openclose” events dropped to only 35.8%.

A 2D node-time (NT) CNN treats frequencies as independent channels, mapping individual frequency bins to the channel dimension, which disrupts the topological proximity between adjacent frequency bands. Reflecting this structural loss, the alternative encoder suffered a severe performance degradation, with the overall macro-averaged F1-score plummeting to 46.0% and the “openclose” F1-score collapsing to just 0.05%.

The proposed 2D FT CNN preserves the unbroken spectrogram grid, enabling the model to extract simultaneous changes in pitch and duration. Conserving this 2D integrity allows the network to capture subtle, high-frequency transients before node-to-node aggregation. This front-end demonstrated the best performance, providing the richest local representation to the dynamic graph generator; it achieved 94.0% accuracy and a macro-averaged F1-score of 86.6%, and also maintained an F1-score of 55.7% on the same sparse “openclose” data.

### 7.4. Sensitivity to the Number of Adjacent Sensor Channels

The spatial context window directly determines the number of graph-adjacent sensor channels. Table 6 details the classification performance, real-time factor (RTF), and floating-point operations (FLOPs) for spatial window sizes expanding from 9 to 21 sensors. Expanding the window initially helps the model capture the complete physical footprint of propagating acoustic waves; consequently, performance exhibits a clear positive correlation with the number of channels up to a specific threshold, with the macro-averaged F1-score peaking at 86.6% using a 17-channel configuration. This structural evolution maps the operational friction between enhancing accuracy and minimizing computational latency. Larger spatial context windows secure higher-fidelity representations, but processing time increases linearly.

Notably, the 17-sensor setup was chosen as the default configuration for this study, as it provides a favorable trade-off between classification accuracy and computational complexity. Scaling up to 17 sensors requires additional FLOPs compared to the smaller window sizes, yet it delivers a substantial improvement in the macro-averaged F1-score, achieving 86.6%, while maintaining the processing time well within the limits for real-time operation. Oversized context windows, such as those in the 21-sensor setup, introduce a critical localization problem: the network may recognize the broad event type perfectly, but the physical epicenter becomes blurred across the expanded receptive field; consequently, the system loses the ability to isolate the specific node of origin. Therefore, expanding the window beyond 17 sensors yields diminishing returns, with further expansion triggering marginal performance degradation. The marginal decline in the macro-averaged F1-score to 85.0% under the 21-sensor setup can be directly attributed to the emergence of spatial oversmoothing. The expanded receptive field inadvertently integrates physically distant, uncorrelated ambient noise, slightly masking localized transient signatures. All tested configurations strictly maintained an RTF below the 0.005 threshold, ensuring continuous real-time execution across all scales.

### 7.5. Impact of Number of Attention Heads

The multi-head attention mechanism generates the dynamic adjacency matrix. Table 7 presents the classification metrics for varying numbers of attention heads. A solitary attention head recorded an 82.2% macro-averaged F1-score, but transitioning to a two-head topology refined the predictions, culminating in an 86.6% macro-averaged F1 and a 94.0% accuracy. Such a bipartite structure inherently allows the model to lock onto multiple distinct physical signatures in parallel.

Increasing the number of heads to four reduced efficiency, and performance decreased slightly in both accuracy and macro-averaged F1-score. These changes appear to stem from redundant features within the expanded attention space, as the model begins to memorize local noise patterns owing to excessive representational capacity. In addition, calculating four separate attention matrices increases the computational cost of the dynamic graph generator. The architecture therefore adopts the two-head configuration, which secures the optimal balance between representational capacity and computational efficiency.

**Figure 6:**
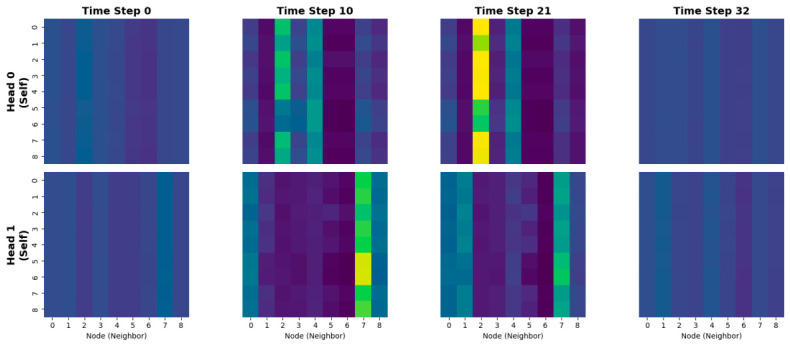
Spatiotemporal visualization of the dynamic adjacency matrices generated by the two independent attention heads. The *x*- and *y*-axis represent the nine spatial neighbors and the central sensor node, respectively. Bright vertical bands indicate strong attention weights, while dark regions denote suppressed channels.

Figure 6 illustrates this representational diversity. The heatmaps map attention weights across 32 continuous time steps for both heads, explicitly exhibiting distinct graph topologies. Notably, the attention weights frequently form distinct vertical columns; this column-wise activation physically signifies that the entire sensor array dynamically routes its focus toward a specific epicenter node where a transient acoustic event is localized. The continuous temporal shifting of these high-weight columns confirms the frame-by-frame dynamic recalculation. Furthermore, the extensive dark regions across the matrices demonstrate active suppression of uninformative noise channels. Ultimately, the attention mechanism enforces structural sparsity while localizing the prominent feature nodes corresponding to the spatial origin of the signal.

### 7.6. Impact of Parameter α in Combination with the Dynamic and Static Graphs

To evaluate the impact of balancing the purely data-driven attention mechanism with the static physical prior, we conducted a sensitivity analysis on the hyperparameter α. In the proposed S2-DyGNN architecture, this parameter dictates the blending ratio between the dynamic adjacency matrix and the fixed physical distance matrix during the graph-generation phase. Table 8 summarizes the classification performance as the value of α varies from 0.1 to 0.3.

As shown in Table 8, the optimal balance is achieved at α=0.15, where the model records a peak overall accuracy of 94.0% and a macro-averaged F1-score of 86.6%. When α is set too low (e.g., 0.1), the influence of the physical distance matrix is insufficient to effectively penalize spurious spatial connections; this lack of structural regularization leaves the dynamic attention mechanism overly susceptible to distant background noise, causing the macro-averaged F1-score to drop to 84.8%.

Conversely, as the value of α increases beyond the optimal threshold (from 0.2 to 0.3), the classification performance exhibits a steady decline, with the macro-averaged F1-score dropping to 85.4% at α=0.3. This degradation occurs because an excessively high α forces the model to rely too heavily on the fixed physical distances; such a rigid topology suppresses the network’s ability to adapt its receptive field dynamically in response to sudden, localized transient acoustic flows. Therefore, maintaining α at 0.15 provides the most effective structural regularization, ensuring that the model captures vital dynamic features without being overshadowed by uncorrelated environmental noise.

### 7.7. Qualitative Analysis of Feature Representations

**Figure 7:**
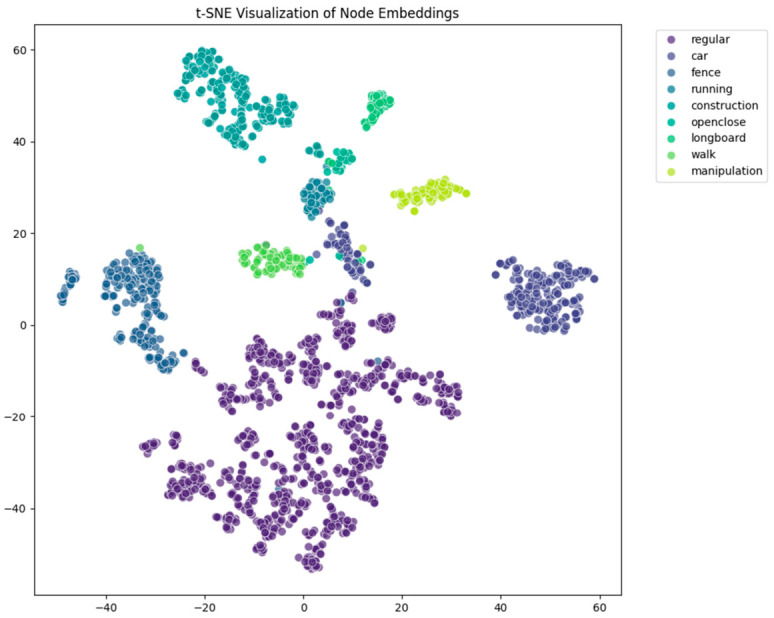
Two-dimensional t-SNE projection of the extracted node embeddings before the final classification layer. The distinct colors correspond to the nine physical acoustic event classes.

To scrutinize the quality of the learned representations, we rendered the high-dimensional node embeddings visually, employing t-distributed stochastic neighbor embedding (t-SNE) to project these latent vectors into a 2D space, thereby validating the extraction capabilities of S2-DyGNN. The model generates these representations immediately before the final classification layer. Figure 7 maps the resulting topology across all nine targeted acoustic categories. The network successfully organized the raw spectrum into linearly separable semantic clusters.

Short-duration signals exhibited minimal variance in the 2D projection. The “running,” “openclose,” and “manipulation” classes condensed into tightly bounded clusters. Short-duration events formed sharply bounded, compact clusters, while continuous signals such as “car” and “regular” presented a highly dispersed spatial distribution, occupying an extended area of the latent space. This expanded spatial distribution is consistent with the continuous acoustic nature, inherent physical variance, and varying durations of these events.

### 7.8. Performance Comparison of the S2-DyGNN and Comparative Models on a Different DAS Dataset

To rigorously evaluate the generalization capability and robustness of the proposed S2-DyGNN architecture across varying hardware configurations and environmental conditions, we conducted additional experiments using a secondary, publicly available Φ-OTDR dataset [42]. This dataset was acquired using a distinct Φ-OTDR setup with an armored sensing fiber deployed in various field scenarios and comprises a total of 15,612 samples that are uniformly distributed across six acoustic events: background noise, digging, knocking, watering, shaking, and walking.

Unlike our primary dataset, which required sliding spatial windows for graph construction, this secondary dataset provides a fixed array of 12 spatial channels per sample, explicitly constraining the graph topology to 12 nodes. Consequently, all 12 available channels were utilized as the spatial context window. To ensure structural compatibility with the proposed architecture, the raw time-series matrices were processed using the exact same STFT protocol applied to the primary dataset.

Following the evaluation protocol established by the dataset authors, the 15,612 samples were strictly partitioned into a training and test set at an 8:2 ratio, ensuring no data overlap. To ensure robust model optimization and prevent overfitting, we further divided the allocated training set into an actual training set and a validation set at an 8:2 ratio. We trained and evaluated the proposed S2-DyGNN along with the baseline models (CNN, LSTM-CNN, GAT, and GCN) on this new dataset. The comparative performance metrics on the unseen test set are presented in Table 9.

As shown in Table 9, the baseline CNN and GCN models struggled to effectively classify the events, yielding macro-averaged F1-scores of 76.8% and 80.3%, respectively. The LSTM-CNN and GAT models demonstrated improved performance, achieving F1-scores of 96.0% and 92.4%, respectively, indicating that capturing temporal dependencies or spatial graph relationships individually provides significant classification benefits on this dataset.

However, the proposed S2-DyGNN consistently outperformed all comparative models, achieving the highest overall accuracy of 97.4% and a macro-averaged F1-score of 97.3%. By dynamically modeling both the intra-channel temporal evolution and the inter-channel spatial wave propagation concurrently, it successfully adapted to the unique noise profiles and spatiotemporal geometries of the new dataset. Despite this smaller neighborhood size compared to the optimized 17-sensor configuration of the primary dataset, S2-DyGNN consistently maintained its performance advantage over the comparative models, demonstrating its architectural robustness under varying spatial scales. These results directly validate the strong generalization capability and structural superiority of the physically regularized dynamic graph approach for DAS event classification, proving its viability beyond a single experimental setup.

### 7.9. Discussion

The experimental results provide explicit validation of the structural necessity of the S2-DyGNN architecture. By integrating a 2D FT CNN with a dual-matrix GNN, the architecture successfully preserves the integrity of localized acoustic features prior to dynamic spatial aggregation. As observed in comparisons with other evaluated models, pure convolutional and graph architectures suffered substantial performance drops on the actual DAS dataset. Regarding specific architectural components, the ablation study on the convolutional front-end empirically demonstrates the critical role of the 2D FT representation. When processing channels independently without explicit 2D spectral mapping, the network struggled to distinguish true transient events from low-frequency baseline shifts, leading to severe degradation in minority class recognition. Implementing the 2D FT CNN bypassed this noise problem: the spectral mapping physically separated target acoustic signatures from background interference, thereby significantly improving minority class accuracy.

The graph topology ablation highlights the operational advantage of the dual-matrix formulation. Because fixed edges fail to expand or contract with evolving acoustic events, strict reliance on static distances severely bottlenecks spatial flexibility; conversely, an unconstrained dynamic graph grants adaptability but risks instability due to an absence of spatial priors. The proposed hybrid approach—fusing data-driven attention with a static distance penalty—achieved the most robust generalization, empirically confirming that dynamic spatial aggregation is most effective when grounded in the physical principles of wave attenuation.

Analysis of attention mechanisms built upon this structural foundation provides key operational insights. Experiments indicated that the dual-head configuration was optimal; increasing the number of heads introduced excessive parameterization, making the network overly sensitive to dataset-specific ambient noise rather than generalizable acoustic features. As visually confirmed by the attention heatmaps, the dual-matrix formulation actively blocks connections with noise-dominated channels. Finally, evaluation of the spatial context window highlights a critical trade-off regarding performance and computational load: while expanding this window initially captures wider physical footprints, indiscriminate overextension incurs severe computational overhead and ultimately leads to spatial oversmoothing. Anchoring the model to an optimal operational boundary resolves this issue. Consequently, the model easily processes 20 kHz streams without latency, making it highly practical for edge-computing hardware.

## 8. Conclusions

Although DAS effectively captures highly nonlinear and complex acoustic wave propagations along fiber-optic cables, detecting localized transient events within continuous streams remains a critical challenge. Raw acoustic signals undergo rapid spatial attenuation and random phase fading, and conventional static networks fail to adapt their topologies to these instantaneous feature variations. To resolve these bottlenecks, the S2-DyGNN architecture was developed in this work, which explicitly integrates a 2D FT CNN with a dual-matrix graph network. The convolutional front-end serves as the primary feature extractor and derives deep spectro-temporal features from each isolated channel, preserving the unbroken geometry of the signal before spatial aggregation. The subsequent graph module fuses data-driven edge formulation with a static physical distance prior. This dynamic spatial aggregation effectively models the shifting feature similarities associated with complex wave propagation along fiber cables, while suppressing irrelevant feature variations from surrounding fiber-optic noise.

When tested against conventional spatial and temporal models, the proposed model showed a clear advantage in processing physical acoustic data: it achieved a peak overall accuracy of 94.0% and a macro-averaged F1-score of 86.6%, representing a 22.5% absolute gain over the foundational CNN baseline. Structural comparative analysis was used to validate the vital role of the proposed 2D FT CNN front-end: compared to a single-axis 1D FT CNN, this dedicated spectro-temporal extractor boosted the overall macro F1-score by 18.2%, confirming its ability to preserve the unbroken 2D geometry of acoustic signals prior to spatial aggregation. Furthermore, in graph topology-specific experiments, the dual-matrix formulation demonstrated the most robust performance on the highly sparse “openclose” anomaly, yielding the highest F1-score of 55.7%, outperforming the rigid static graph topology by a 7.7% margin. Together, these quantitative metrics confirm that the network correctly penalizes spurious spatial connections and prevents spatial oversmoothing. Furthermore, even without using the optimized 17 adjacent channels configuration (e.g., when using 12 adjacent channels), S2-DyGNN consistently maintained its performance advantage over comparative models, demonstrating its robustness to different spatial scales.

In addition to classification accuracy, the proposed architecture displays extreme computational efficiency. When the spatial context window was limited to 17 adjacent channels or fewer, the RTF did not exceed 0.005. These metrics ensure latency-free execution on 20 kHz high-frequency data streams. Owing to this lightweight profile, the model is applicable to real-world edge-computing systems; in particular, it can be seamlessly integrated into on-site early-warning systems for pipeline leak detection in nuclear power plant environments, real-time railway tracking, and perimeter security across critical facilities. Future research will extend this dual-matrix framework to disentangle overlapping acoustic signatures under extreme multisource interference environments.

## Figures and Tables

**Table 1:** Operational parameters of the DAS system.

Parameter	Value
Pulse width	20 ns
Spatial sampling interval	1.02 m
Pulse repetition rate	20 kHz
Maximum detectable acoustic frequency	0–10 kHz

**Table 2:** Number of recorded samples per event scenario.

Event Scenario	Event Count
Car	20,957
Construction	29,169
Climbing	3601
Longboard	11,908
Manipulation	17,223
Openclose	5943
Regular	210,023
Running	12,927
Walking	12,714

**Table 3:** Performance comparison of the conventional event classification models and S2-DyGNN, measured by overall accuracy and macro-averaged F1-scores.

Model	Accuracy (%)	Macro-Averaged F1-Score (%)	Openclose F1-Score (%)
CNN	84.3	64.1	18.8
LSTM-CNN	86.0	71.4	31.2
GAT	81.2	67.3	28.6
GCN	85.3	75.5	37.2
S2-DyGNN (proposed)	94.0	86.6	55.7

**Table 4:** Performance comparison across different graph topology configurations in S2-DyGNN.

Model	Graph Topology	Accuracy (%)	Macro-Averaged F1-Score (%)	Openclose F1-Score (%)
Static GNN-CNN	Physical distance	89.9	78.3	48.0
Dynamic GNN-CNN	Data-driven attention	90.6	85.5	52.3
S2-DyGNN (proposed)	Hybrid (attention + distance penalty)	94.0	86.6	55.7

**Table 5:** Performance comparison across different convolutional front-ends in S2-DyGNN.

CNN Type	Accuracy (%)	Macro-Averaged F1-Score (%)	Openclose F1-Score (%)
1D FT CNN	88.6	68.4	35.8
2D NT CNN	75.3	46.0	0.05
2D FT CNN	94.0	86.6	55.7

**Table 6:** Impact of spatial context window size on classification performance, floating-point operations, and real-time factors.

Number of Adjacent Sensors	Accuracy	Macro-Averaged F1-Score	FLOPs	RTF
9	89.9	80.1	1.14 G	0.002468
13	92.6	83.6	1.65 G	0.003274
17	94.0	86.6	2.16 G	0.004150
21	93.5	85.0	2.67 G	0.004929

**Table 7:** Performance comparison across different multi-head attention configurations.

Number of Heads	Accuracy	Macro-Averaged F1-Score
1	91.8	82.2
2	94.0	86.6
4	92.0	82.6

**Table 8:** Performance comparison across different α configurations.

Value of α	Accuracy	Macro-Averaged F1-Score
0.1	90.3	84.8
0.15	94.0	86.6
0.2	90.8	85.5
0.3	90.2	85.4

**Table 9:** Performance comparison of the conventional event classification models and S2-DyGNN on a different DAS dataset, measured by overall accuracy and macro-averaged F1-scores.

Model	Accuracy (%)	Macro-Averaged F1-Score (%)
CNN	78.5	76.8
LSTM-CNN	96.1	96.0
GAT	92.8	92.4
GCN	80.8	80.3
S2-DyGNN (proposed)	97.4	97.3

## Data Availability

Publicly available datasets were analyzed in this study. This data can be found here: https://doi.org/10.6084/m9.figshare.27004732.v1.
